# Comparison of *Coxiella burnetii* Excretion between Sheep and Goats Naturally Infected with One Cattle-Associated Genotype

**DOI:** 10.3390/pathogens9080652

**Published:** 2020-08-13

**Authors:** Benjamin Bauer, Louise Prüfer, Mathias Walter, Isabel Ganter, Dimitrios Frangoulidis, Martin Runge, Martin Ganter

**Affiliations:** 1 Clinic for Swine and Small Ruminants, Forensic Medicine and Ambulatory Service, University of Veterinary Medicine Hannover, Foundation, Bischofsholer Damm 15, 30173 Hannover, Germany; martin.ganter@tiho-hannover.de; 2 Lower Saxony State Office for Consumer Protection and Food Safety (LAVES), Food and Veterinary Institute Braunschweig/Hannover, Eintrachtweg 17, 30173 Hannover, Germany; Louise.Pruefer@LAVES.Niedersachsen.de (L.P.); Martin.Runge@LAVES.Niedersachsen.de (M.R.); 3 Bundeswehr Institute of Microbiology, Neuherbergstraße 11, 80937 Munich, Germany; mathiaswalter@instmikrobiobw.de (M.W.); DimitriosFrangoulidis@instmikrobiobw.de (D.F.); 4 Department of Psychology, Faculty of Life Sciences, Humboldt-Universität zu Berlin, Unter den Linden 6, 10099 Berlin, Germany; Ganteris@hu-berlin.de; 5 Bundeswehr Medical Service Headquarters VI-2, Medical Intelligence & Information (MI2), Dachauer Straße 128, 80637 Munich, Germany

**Keywords:** *Coxiella burnetii*, sheep, goat, cattle, environmental contamination, nasal swabs, whole genome sequencing, MLVA/VNTR, Q fever, zoonosis

## Abstract

The main reservoir of *Coxiella (C.) burnetii* are ruminants. They shed the pathogen through birth products, vaginal mucus, faeces and milk. A direct comparison of *C. burnetii* excretions between naturally infected sheep and goats was performed on the same farm to investigate species-specific differences. The animals were vaccinated with an inactivated *C. burnetii* phase I vaccine at the beginning of the study period for public health reasons. Vaginal and rectal swabs along with milk specimens were taken monthly during the lambing period and once again at the next lambing season. To estimate the environmental contamination of the animals’ housings, nasal swabs from every animal were taken simultaneously. Moreover, dust samples from the windowsills and straw beddings were collected. All samples were examined by qPCR targeting the IS*1111* gene and the MLVA/VNTR typing method was performed. Whole genome sequencing was applied to determine the number of IS*1111* copies followed by a calculation of *C. burnetii* genome equivalents of each sample. The cattle-associated genotype C7 was detected containing 29 IS*1111* copies. Overall, goats seem to shed more *C. burnetii* through vaginal mucus and in particular shed more and for longer via the rectal route than sheep. This is supported by the larger quantities of *C. burnetii* DNA detected in caprine nasal swabs and environmental samples compared to the ovine ones. Transmission of *C. burnetii* from cattle to small ruminants must also be considered.

## 1. Introduction

*Coxiella burnetii* is an intracellular bacterium with a high zoonotic potential causing serious health problems, like pneumonia and endocarditis in humans [1]. Infected mammals shed large amounts of the pathogen during abortion or normal delivery through birth products. Additionally, the pathogen is shed through vaginal mucus, faeces and milk for several months [2,3,4]. The highly contagious pathogen infects animals and humans particularly through the inhalation of contaminated aerosols or dust and no direct contact to shedders is necessary. Sheep and goats are the main reservoirs for the pathogen and the herd prevalence is over 30% in several countries [5,6,7]. Consequently, small ruminants are the major source for many human Q fever epidemics in Europe [8]. For instance, one lambing ewe infected almost 300 visitors to a German farmers’ market [9]. In the Netherlands, several abortion waves on dairy goat farms were responsible for the largest human Q fever outbreak worldwide [10]. Cattle are also affected by the pathogen, but this ruminant species seems to play a minor role as a source of human epidemics [8,11]. The shedding duration and quantity of *C. burnetii* is described for each ruminant species separately [2,3,4]. In general, goats shed the pathogen through vaginal mucus, faeces and milk [12]. In contrast, sheep excrete less in milk but larger amounts through vaginal mucus and faeces [12]. The main route of shedding in cattle is via milk and to a lesser extent through vaginal mucus [12]. Cattle shed very little *C. burnetii* through faeces [4,12]. Excretion of the pathogen in the next lambing season was described for small ruminants [13,14]. However, direct comparisons between ruminant species kept under the same environmental conditions and infected with the same *C. burnetii* genotype are missing. 

An inactivated *C. burnetii* phase I vaccine is licensed in several European countries for cattle and goats to prevent *C. burnetii* shedding [15]. In the past, this vaccine has also been applied to sheep [13,16,17]. In case of a Q fever outbreak, vaccination of small ruminants is recommended but is less effective than preventive vaccination [18]. In infected flocks, vaccination reduces the shedding over an extended period and should therefore be implemented as a long-term control measure [13,18,19]. 

Due to the extensive excretion of *C. burnetii* from small ruminants during a Q fever outbreak, their housings are highly contaminated with the pathogen and are a potential risk for humans conducting farm visits [20,21]. Air samples were used to estimate the amount of *C. burnetii* in the air of barns [2,21,22]. Furthermore, swab cloths and petri dishes were used to collect dust from windowsills and fences to detect and monitor *C.-burnetii*-positive ruminant husbandries [2,23]. *C. burnetii* DNA can be detected in environmental samples for years on farms after a Q fever outbreak [13]. However, the detection of *C. burnetii* DNA fragments in the environment gives neither information about the current excretion status of the animals nor about the viability and infectivity of the pathogen.

The IS*1111* insertion sequence, coding for a transposase, contains between seven and 110 copies in *C. burnetii* genomes [24,25]. Therefore, this element is widely used as a specific target providing sensitive diagnostic PCRs [24]. However, accurate calculation of the *C. burnetii* quantity in samples is difficult to achieve with this multicopy insertion sequence. A quantitative PCR method with the target IS*1111* has been used in combination with calibrated standards prepared from the Nine Mile RSA493 isolate to quantify the amount of genome equivalents (GE) per sample [2,23,25,26]. Additionally, due to the growing number of whole genome sequences from *C. burnetii*, the numbers of IS*1111* copies of an isolate could be extracted directly from the sequence data via bioinformatics analysis.

In the past, different molecular techniques have been used to characterize *C. burnetii* and the different approaches were recently summarized by Massung et al. [24]. The multiple locus variable number tandem repeats analysis (MLVA/VNTR) has a higher discrimination power than the Multispacer-Sequence-Typing (MST) method [24,27]. The MLVA/VNTR uses up to 17 different genomic target regions for the differentiation of *C. burnetii* strains. Several genotypes could be characterized within the ruminant population across Europe [28,29,30,31]. Nevertheless, using different markers and a variable number of them hamper the comparison. Recently, three main clusters were identified: Cluster A and B were mainly associated with small ruminants, whereas cluster C was mostly found in samples from bovine origin [32,33]. This supports the assumption about host specificity of single *C. burnetii* strains [34]. Of note, different genotypes can circulate within the same farm at the same time [30,33,35]. 

In the present study, the excretion of *C. burnetii* in naturally infected sheep and goats was compared on the same farm for two successive lambing seasons. For that purpose, different sample matrices were taken from the small ruminants. Moreover, a novel approach was applied to estimate the environmental contamination of *C. burnetii* of the small ruminants’ housings by testing nasal swabs of all sheep and goats [36]. To underline these results, samples from the barns’ dust and straw bedding were collected including the cattle on the farm. The estimations of *C. burnetii* concentrations were based on the calculation of *C. burnetii* quantity by real time quantitative PCR and based on the IS*1111* copies determined by whole genome sequencing. Furthermore, to examine the epidemiological background of the *C. burnetii* infection, MLVA/VNTR typing was performed from samples collected from sheep, goats and cattle. For public health reasons, all ruminants were vaccinated twice with an inactivated *C. burnetii* vaccine at the beginning of the study period.

## 2. Results

The study included 37 sheep, 22 goats and five cattle kept on the same farm. Every ruminant species was housed in separate stables located side by side, and the animals of the different species never had direct contact either during the housing period nor on the pastures. The stocking densities in the sheep and goat barn were similar. It was always the same animals that were sampled with different kinds of sample matrices for two successive lambing seasons. No new livestock were brought onto the farm during the entire investigation period. Moreover, no biosecurity measures were implemented by the animal owner despite the detection of *C. burnetii* in his animals.

In total, 182 vaginal swabs, 183 rectal and nasal swabs, respectively, as well as 92 milk specimens were analyzed from sheep. Furthermore, in each case 94 vaginal, rectal and nasal swabs and 37 milk specimens from goats were examined for *C. burnetii* DNA. Additionally, 22 vaginal swabs from cattle were analyzed. Ten dust swabs from the windowsills and ten samples from the straw bedding were also taken from every ruminant housing. However, only nine straw bedding samples were taken from the cattle barn.

The MLVA/VNTR typing revealed the cattle-associated C7 genotype in samples from all three ruminant species and the whole genome sequence analysis of one strain identified 29 copies of the IS*1111* element.

**Figure 1:**
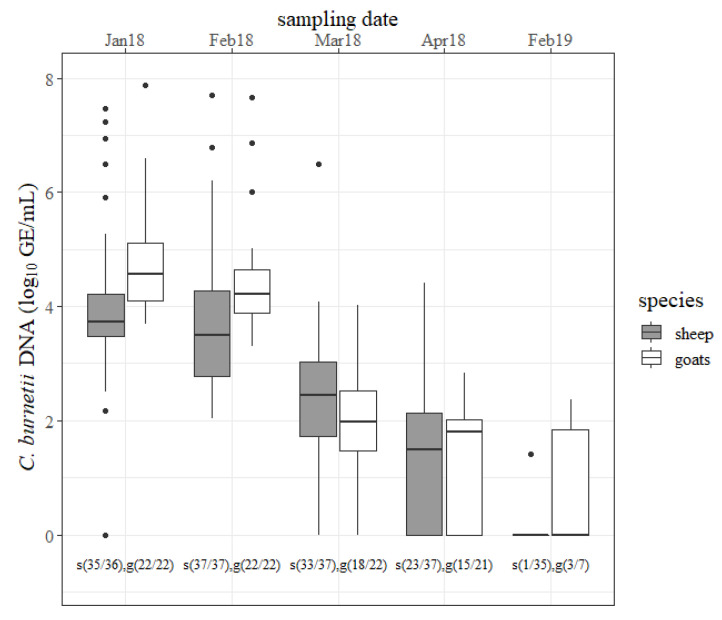
Quantity of *Coxiella burnetii* DNA (log_10_ GE/mL) determined on vaginal swabs from sheep (gray) and goats (white) for each sampling date (month/year). s = sheep, g = goats, and the number in brackets = the number of positive tested vaginal swabs/number of examined vaginal swabs.

The results of the vaginal swabs are shown in Figure 1. During the first two months of the study period, a quantity of ˃6 log *C. burnetii* GE/mL per swab was detected in seven different ewes. The concentration of *C. burnetii* detected with ovine vaginal swabs declined but only the decrease from February to March 2018 was significant (*p* < 0.05). At the next lambing season, only one ewe shed the pathogen (<2 log GE/mL) via the vaginal route. In five caprine vaginal swabs, *C. burnetii* amounts of ˃6 log GE/mL from different does were determined in January 2018 and February 2018. The amount of the pathogen decreased from one sampling date to the next in 2018 but only the decline from February 2018 to March 2018 was significant (*p* < 0.05). At the next lambing season, three goats still shed *C. burnetii* through vaginal mucus (<3 log GE/mL). In the first two months, goats shed more *C. burnetii* through vaginal mucus than sheep (*p* < 0.05). The number of positive vaginal swabs was not significantly different between sheep and goats at any sampling date in 2018 but more goats than sheep shed the pathogen through vaginal mucus at the next lambing season (*p* < 0.05).

**Figure 2:**
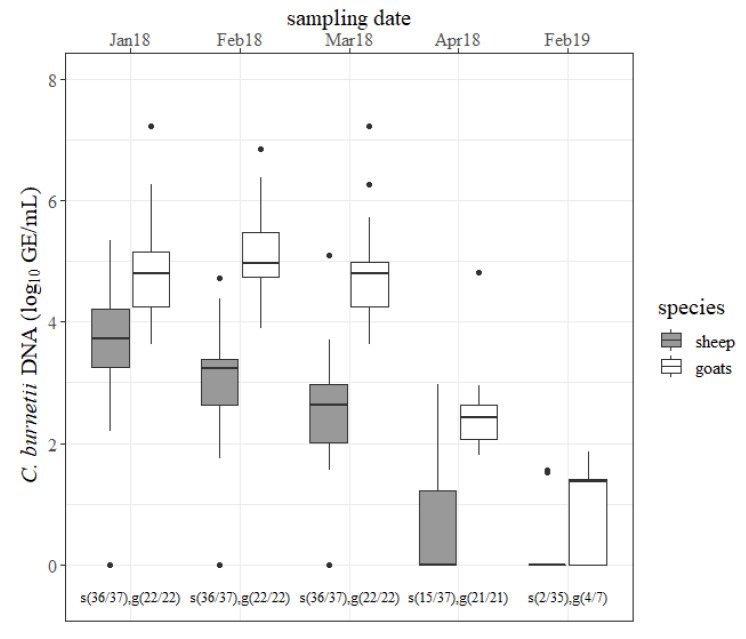
Quantity of *Coxiella burnetii* DNA (log_10_ GE/mL) determined on rectal swabs from sheep (gray) and goats (white) for each sampling date (month/year). s = sheep, g = goats, and the number in brackets = the number of positive tested rectal swabs/number of examined rectal swabs.

The outcomes of the rectal swabs are presented in Figure 2. Ovine rectal shedding decreased from one to the next sampling date in 2018. The declines from January to February 2018 and from March to April 2018 were significant (*p* < 0.05). Only two ewes shed the pathogen (<2 log GE/mL) via the rectal route in 2019. In goats, an amount of *C. burnetii* of ˃6 log GE/mL was determined from rectal swabs from six different does at the first three sampling dates. Moreover, the quantity of *C. burnetii* from rectal swabs from goats stayed at a high level followed by a strong decline in April 2018 (*p* < 0.05). At the next lambing season, still more than half of the goats were shedding the pathogen via the rectal route. In 2018, goats shed more *C. burnetii* via the rectal route than sheep at any sampling date (*p* < 0.05). More *C.*-*burnetii*-positive rectal swabs were detected from goats than from sheep in April 2018 and February 2019 (*p* < 0.05). 

**Figure 3:**
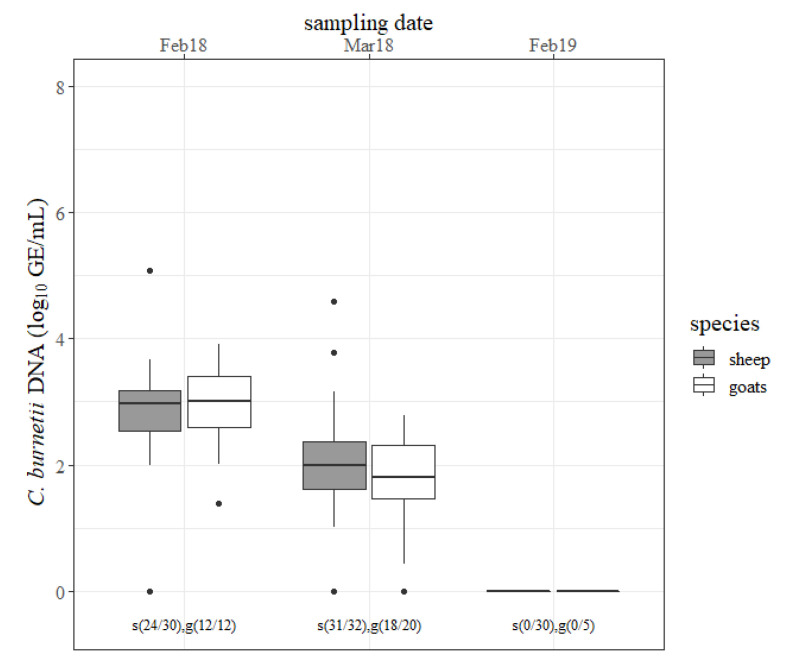
Quantity of *Coxiella burnetii* DNA (log_10_ GE/mL) determined in milk specimens from sheep (gray) and goats (white) for each sampling date (month/year). s = sheep, g = goats, and the number in brackets = the number of positive tested milk specimens/number of examined milk specimens.

The results of the milk specimens are outlined in Figure 3. Most of the involved animals shed *C. burnetii* through milk in February 2018 and March 2018 and no small ruminant shed the pathogen through milk at the next lambing season. The amount of detected *C. burnetii* in milk decreased (*p* < 0.05) from February 2018 to March 2018 in both species. There was no difference in the concentration of *C. burnetii* in milk and the numbers of shedders between sheep and goats at any sampling date.

**Figure 4:**
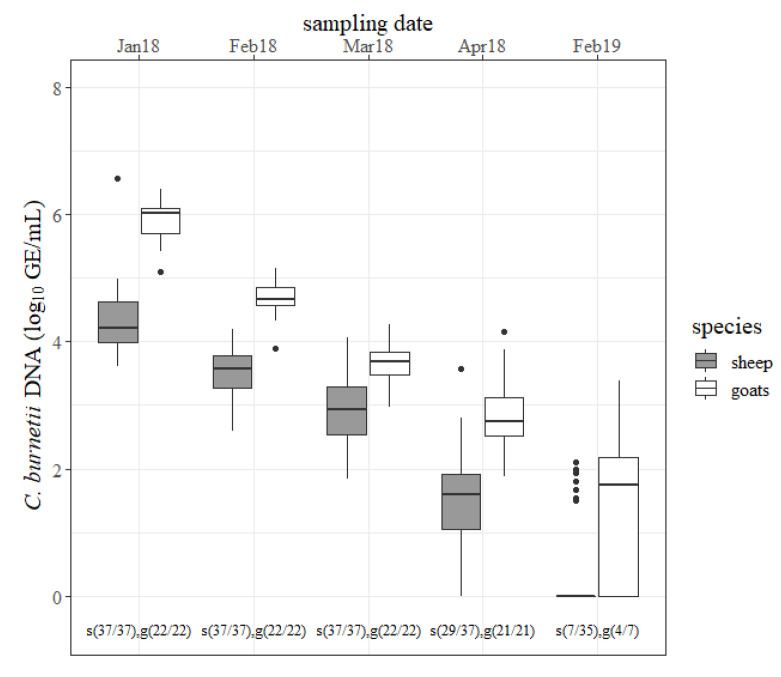
Quantity of *Coxiella burnetii* DNA (log_10_ GE/mL) determined on nasal swabs from sheep (gray) and goats (white) for each sampling date (month/year). s = sheep, g = goats, and the number in brackets = the number of positive tested nasal swabs/number of examined nasal swabs.

The outcomes of the nasal swabs are presented in Figure 4. In January 2018, most of the caprine nasal swabs contained ˃ 6 log GE/mL of *C. burnetii*, whereas only one nasal swab from sheep was detected with such a large amount. The quantity of *C. burnetii* from ovine swabs decreased constantly from one sample date to the other (*p* < 0.05). Like sheep, the concentration of *C. burnetii* from caprine nasal swabs also declined continuously (*p* < 0.05). The number of positive nasal swabs was not significantly different between sheep and goats at any sampling date. Several nasal swabs from sheep and goats tested *C. burnetii* positive at the 2019 lambing season but the pathogen’s concentration was less compared to the previous year. Nasal swabs from goats contained more *C. burnetii* than from sheep at any sampling date in 2018. The differences in January 2018, February 2018 and April 2018 were significant (*p* < 0.05).

During the 2018 lambing period, goats shed more *C. burnetii* from February to April via the rectal route than in the vaginal mucus (*p* < 0.05). In sheep, the quantity of *C. burnetii* shed via the vaginal and rectal routes was not significantly different at any sampling date in 2018. Finally, in the 2019 lambing season, the number of positive sheep and goats and the quantity of *C. burnetii* on the swabs and milk specimens were considerably less compared to the previous year. 

The five cattle were sampled on the same dates as the small ruminants. During the study period January until April 2018 two cows calved on the same day in March and vaginal swabs were taken nine days after calving. These samples contained between 1 log GE/mL and 6 log GE/mL. The other three cows were pregnant during that time. From January to March 2018 all cows had positive vaginal swabs (1 log < 6 log GE/mL), but all cows were tested negative in April 2018. In February 2019, only two non-pregnant cows were tested and both animals were negative. 

**Figure 5:**
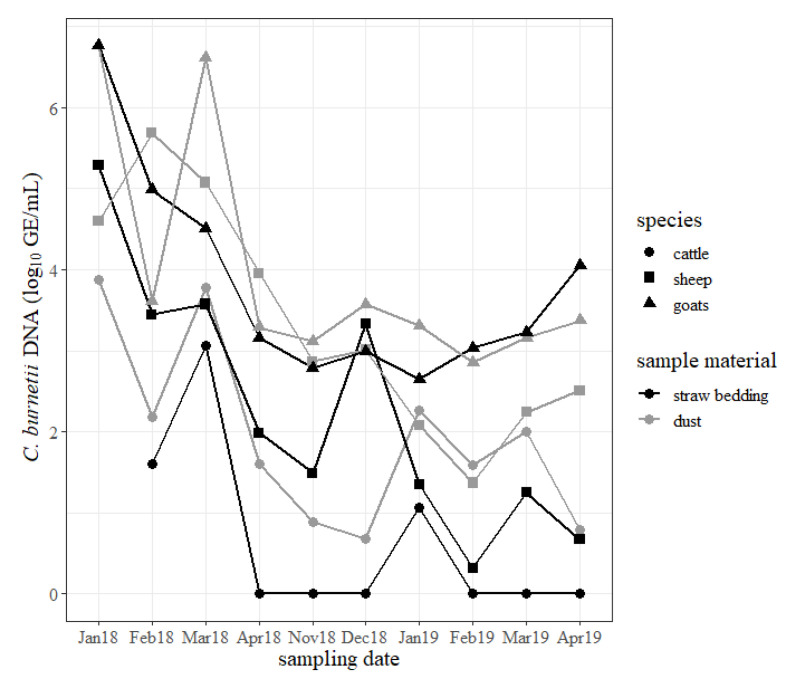
Quantity of *Coxiella burnetii* DNA (log_10_ GE/mL) determined on the swabs from the windowsills (dust) of cattle ●, sheep ■ and goat ▲ barns (gray) and on the straw bedding swabs (black) from all three ruminant species for each sampling date (month/year). The bovine straw bedding sample from January 2018 is missing.

The results of the dust and straw bedding samples are shown in Figure 5. During the study period, dust and straw bedding samples from the goat shed mostly revealed the highest concentration of *C. burnetii* compared to samples from the sheep shed and cattle barn. Specifically, the samples from the cattle barn generally included the lowest quantity of the pathogen. Independently from the animal housings, specimens from the windowsills usually contained more *C. burnetii* than samples taken from the straw bedding. The concentration of *C. burnetii* in swabs from dust and straw bedding from the sheep and goat sheds decreased but stayed positive for *C. burnetii* DNA until the end of the study. The concentration of *C. burnetii* DNA in the dust and straw bedding from the cattle barn increased briefly in March 2018 after two cows shed *C. burnetii* during calving, but the rise in January 2019 cannot be explained by such an event. From April 2018 until April 2019 most of the samples from the straw bedding from the cattle barn tested negative for *C. burnetii* DNA.

## 3. Discussion

The present study focused on the differences in *C. burnetii* shedding between sheep and goats naturally infected with the same genotype of *C. burnetii* and kept indoors under the same conditions. The results were underlined by the estimation of *C. burnetii* contamination of the environment using the novel indirect approach of nasal swabs from the animals and direct measuring with swabs from straw beddings and dust from the windowsills. Furthermore, whole genome sequencing identified one strain with 29 copies of IS*1111* elements. This result was used to estimate the quantity of *C. burnetii* genome equivalents for each collected sample. This approach has been not widely used yet. Most authors are using, for instance, a quantitative PCR with target sequence *com1* or calibrated standards from *C. burnetii* strain Nine Mile RSA 493 isolate to estimate the amount of the pathogen [2,3,23,25,26]. Therefore, a direct comparison of the quantity of *C. burnetii* with results from other studies is hampered due to the different methods used. In general, the amount of excreted *C. burnetii* declined during the study period in both species. This is in accordance with previous studies and reflects the natural shedding behavior of *C. burnetii* in small ruminants [2,3,21,37]. In the past, shedding routes of *C. burnetii* were compared between sheep, goats and cattle but without pathogen quantification [12]. Moreover, this comparison was conducted between several pure sheep, goat and cattle farms with undefined genotypes and kept under different environmental and management conditions.

Single animals shed large amounts of *C. burnetii* confirming the assumption of super-spreaders within infected flocks [2,9]. At the beginning of the study, goats shed significantly larger amounts of *C. burnetii* through vaginal mucus than sheep. Additionally, more goats than sheep shed the pathogen at the next lambing season. This could be the reason for the huge Q fever outbreak in the Netherlands from 2007 until 2011. Sheep played a minor role as a cause of human Q fever infection during that time, although the Dutch sheep population is larger than the goat population [10,38]. Furthermore, the big dairy goat farms are using synchronization programs followed by artificial insemination to concentrate kidding to a very short period [39]. Consequently, many infected goats gave birth simultaneously and this led to the release of tremendous amounts of *C. burnetii* into the environment. In contrast, synchronization and artificial insemination are less commonly used in the sheep industry and lambing periods are more expanded with less ewes lambing simultaneously and probably a smaller release of *C. burnetii* at the same time. 

In the present study, goats shed significantly more *C. burnetii* via the rectal route than sheep. Moreover, significantly more positive rectal swabs were detected from goats than from sheep indicating a longer period of excretion by goats. The amount of *C. burnetii* shed by the rectal route was higher than by the vaginal route in goats. These findings might also be the reason for the Dutch Q fever epidemic as mentioned above. Rectal excretion of *C. burnetii* in small ruminants was described by several authors [2,17,21,40]. However, this route of shedding is still under discussion. Roest et al. [37] could not find any indication of active replication of the pathogen either in the gut nor in the liver or in the bile duct of experimentally infected goats. These authors detected *C. burnetii* in faeces after the first goat aborted in the study group. Therefore, Roest et al. [37] concluded that positive faecal samples occur more from a contaminated environment than by active shedding. In case of an outbreak, small ruminants ingest huge amounts of the pathogen with their daily food intake. Therefore, it could be possible that *C. burnetii* only pass through the digestive tract leading to positive rectal swabs and faecal samples. In contrast, Arricau-Bouvery et al. [40] detected *C. burnetii* in caprine faeces after 25 days of subcutaneous infection and just before the goats aborted. In a recently published study, Sobotta et al. [41] infected bovine epithelial cells from the intestine with *C. burnetii*. The intestinal epithelial cells allowed moderate invasion of the pathogen but there was little further propagation of bacterial numbers within these cells. This observation is in line with the findings from in vivo studies showing that cattle shed to a lower extent *C. burnetii* in faeces [4,12]. The application of this study design to intestinal epithelial cells originating from sheep or goats could reveal contradicting results, due to possible species-specific responses to *C. burnetii*. Manure from *C.*-*burnetii*-positive sheep and goat farms are a major source of human Q fever infections [8,11]. Moreover, rectal swabs from goats contained significantly more *C. burnetii* than vaginal swabs in the present study. It is unlikely that this large amount of *C. burnetii* bacteria in the faeces can be explained alone by ingestion and passage through the gastro-intestinal tract (GIT). Specifically, the concentrations on the goats’ rectal swabs in comparison with the vaginal shedding indicates an additional active multiplying of the bacterium in the GIT. This needs further clarification.

The collection of milk specimens from every small ruminant was not always possible during the entire study period because the sheep and goats are non-dairy breeds. There was no significant difference between sheep and goats regarding excretion of *C. burnetii* in milk. The quantity of *C. burnetii* was less compared to the amount in vaginal and rectal swabs. This is in accordance to other studies finding low burdens of the pathogen in milk compared to the other excretion routes [2,3,21,42]. At the next lambing season, neither sheep nor goats shed *C. burnetii* through milk. 

In the past, nasal swabs were not widely used to detect *C. burnetii* [36,43]. In the current study, we used this method to estimate the contamination of *C. burnetii* in the inhaled air of the sheep and goats. This approach does not need any technical support, and a consistent filtering of tidal air occurs through the nasal cavity of the animals, imitating the natural route of infection. The three ruminant species lived in different closed barns on the same farm. The estimated numbers of *C. burnetii* per swab were significantly larger in goats than in sheep. Therefore, we concluded that the goat shed is more heavily contaminated with *C. burnetii* compared to the sheep shed due to the greater caprine excretion rate. This finding is supported by the results of the dust and straw bedding swabs with the largest amount found in the goat barn during the entire study period.

It is important to bear in mind that a direct comparison of small ruminants and cattle is difficult to apply. Small ruminants have a dense lambing period, shedding large amounts of *C. burnetii* within a few weeks. In contrast, single cows are calving and shedding during the entire year. This could explain the smaller quantities of *C. burnetii* in the bovine environment compared to small ruminant farms which was also reported by Carrié et al. [23]. Furthermore, small ruminants excrete massive amounts of *C. burnetii* through the vaginal and rectal routes, whereas cows shed the pathogen through milk and less frequently through vaginal mucous and faeces [12]. Therefore, environmental contamination and the risk for human infections seems to be larger in *C.-burnetii*-positive small ruminant husbandries than in positive cattle farms. Environmental samples stayed positive during the whole study period, despite straw bedding being removed but neither cleansing nor disinfection were applied. This is in line with other studies. *C. burnetii* DNA is detectable in animals’ housings after an outbreak for several months [2,16,20]. However, the ability of infectivity of low quantities of *C. burnetii* on infected farms is still doubtful. For that purpose, the intraperitoneal inoculation of BALB/c mice, or the housing of guinea pigs in the ruminant barns would be necessary, which is difficult to conduct due to ethical and technical reasons [20,21]. A viability of *C. burnetii* in dust samples of up to two months after the last abortion has been reported [21]. The results from surface swabs of windowsills and barn fences correlated with the excretion of *C. burnetii* [44]. This is consistent with our findings. Dust and straw bedding samples contained the largest amounts of *C. burnetii* at the beginning of the abortion wave and during the lambing period in 2018 when vaginal and rectal shedding was also the highest. Moreover, a short peak in bovine dust and straw bedding samples was observed in March 2018, detected nine days after two *C.*-*burnetii*-positive cows calved. Further long-term investigations are necessary to evaluate and standardize the application of surface swab samples as an economic monitoring tool for ruminant husbandries. This would include the establishment of reliable thresholds that would indicate an acute Q fever outbreak. Consequently, an active surveillance program for non-dairy ruminants could be implemented, which is absolutely necessary because of the missing application of bulk tank milk samples.

We were able to identify the cattle-associated C7 genotype in vaginal swabs from sheep, goat and cattle on the same farm, although the three species did not share the same barn. The C7 genotype was so far only seen in a cattle herd also located in southern Germany [32]. In the past, cluster C was rarely detected in samples from small ruminants with no information about the occurrence of cattle on the same property [32,33]. Moreover, genotypes from cluster A and B were hardly detected in cattle and were mainly reported in areas with a high density of small ruminants [32,33]. Consequently, cross infection within the ruminant population occurs but less is known regarding susceptibility to single genotypes within the ruminant population. In the present investigation, we can only speculate about the transmission of the C7 genotype from the farm-owned cattle herd to the sheep and goat flocks. Nevertheless, asymptomatic cattle herds must be considered as potential reservoirs capable of transmitting *C. burnetii* to other animal species [42]. The identification of genotypes still gives no information about its pathogenicity or virulence. In the current study, only the goats aborted, with 17 out of 22 goats having abortions, and sheep and cows did not show any reproductive disorders and healthy lambs and calves were born. Therefore, further research should be performed to investigate the species and organ-specific response and outcomes of specific *C. burnetii* genotypes within the different ruminant species.

The authors are aware of the limitations of this field study. Firstly, on the basis of the recommendation of the European Food Safety Authority (EFSA) [18], all ruminants were vaccinated at the beginning of the study period to reduce the risk of the local residents of the village being infected in the future. The vaccination of *C.*-*burnetii*-infected small ruminants led to contradictory results of *C. burnetii* shedding [45]. However, it is a common consensus that vaccination against *C. burnetii* has rather a long-term impact on the pathogen’s excretion than preventing from shedding during an active outbreak [16,18,19]. The reasonable lack of unvaccinated control animals makes a statement about the efficiency of the vaccine unfeasible. Moreover, we cannot rule out any differences in vaccine responsiveness between sheep and goats with a possible effect on the shedding behavior. In the past, the shedding kinetics of vaccinated and unvaccinated small ruminants were described separately for each species [2,3,14,37]. Their variance regarding study design, methods and enviromental conditions make a comparison difficult. Further studies are needed to confirm our findings with unvaccinated sheep and goats under controlled experimental conditions. Nevertheless, the focus of the present study was not to evaluate the vaccine. The main purpose was a direct comparison of *C. burnetii* excretion between sheep and goats naturally infected with the same genotype of the pathogen and within an epidemiological unit in order to get deeper insights into the complexity of *C. burnetii* infection within different ruminant species. Hermans et al. [46] reported that vaccine-derived *C. burnetii* DNA was excreted in milk up to 9 days after vaccination. This has not been evaluated for other shedding routes, yet [45]. In the current study, sampling was conducted three and four weeks after vaccination. Moreover, no vaccination was applied in 2019. Therefore, we assume that vaccine-derived *C. burnetii* DNA was not detected through any excretion route at any sampling date. 

Sampling mucosal-like tissue from the vagina, rectum or nasal cavity with swabs is a simple method to obtain sample material for the detection of *C. burnetii*. However, this sampling technique has its limitations. The amount of absorbed secretions and pathogens on a swab is difficult to standardize and a quantitative comparison is therefore hampered. Consequently, the calculated *C. burnetii* genome equivalents of each swab in the present study is a rough assessment. This also applies for the dust and straw bedding samples. Therefore, the authors decided to forego the mention of *C. burnetii* concentration in absolute figures. Our intention was to standardize the sampling procedures as far as possible under field conditions and to use a sampling tool that can be easily and safely processed in the laboratory to reduce the infection risk for technicians. Therefore, we used the same dry viscose swab for all sampling matrices. Both the excretion of *C. burnetii* and environmental contamination decreased during the study period, which is consistent with previous findings [2,3,21,37]. Therefore, the applied sampling technique appears to be appropriate and a comparison between ruminant species is possible.

Unfortunately, in the next lambing season, only seven goats were left from 2018. This sample size is small and limits the explanatory power in 2019. Moreover, the number of cattle is small and the breeding and management conditions are different compared to the small ruminants. However, these differences reflect the common livestock situation in southern Germany. Therefore, the results of dust and straw bedding from the cattle barn just gives an indication about the quantity of *C. burnetii* in the bovine environment and a comparison with the outcomes from the small ruminants is hampered.

## 4. Conclusions

Overall, goats seem to shed significantly more *C. burnetii* in vaginal mucus and significantly more and for a longer period via the rectal route than sheep. This is supported by the greater environmental contamination with *C. burnetii* in the goat barn based on the results from the nasal swabs, windowsill and straw bedding samples. Moreover, dust swabs from the surfaces of stables can be a useful and affordable tool to detect *C.-burnetii*-positive livestock. Finally, a cattle-associated genotype can circulate within the ruminant population, leading to a high abortion rate in goats but causing no obvious clinical problems in sheep and cattle. Further research is necessary to confirm our observations. Specifically, the species-specific differences have to be investigated under controlled experimental conditions.

## 5. Material and Methods

### 5.1. Animals and Sampling Procedure

In January 2018, *C. burnetii* was diagnosed from aborted material (placenta and foetus) from a goat on a hobby farm. This farm included 22 goats (German Fawn Improved x Boer Goat), 37 sheep (Coburger Fox) and 5 cows (Hinterwald cattle). Every ruminant species was housed in separate stables and never had direct contact either during the housing nor pasture period. However, the stables were located side by side and the farmer used the same equipment. The lambing season in goats and sheep took place from 10th January 2018 until 12th February 2018 and from 16th January 2019 to 10th February 2019. From January 2018 until April 2018 and in February 2019 all small ruminants were professionally restrained and were sampled with dry sterile swabs consisting of a small viscose swab on a 9 cm polystyrene stick (Sarstedt, Nümbrecht, Germany). The vaginal and rectal swabs were taken by introducing the swab for approximately 7 cm into the vagina or rectum. Then, the swab was turned lightly for 10 s. These samples were collected every three to four weeks to determine the quantity and duration of *C. burnetii* excretion. For the same purpose, sterile milk specimens were taken from each half of the udder but collected in one sterile tube (Sarstedt, Nümbrecht, Germany). However, milk samples could not be collected from every animal at every single sample date because the animals were non-dairy breeds. Simultaneously, nasal swabs (Sarstedt, Nümbrecht, Germany) were collected to estimate the environmental contamination of the sheep and goat sheds. The head of each restrained animal was held and a sterile swab was introduced for approximately 7 cm into the left nasal cavity. The nasal mucus was sampled by lightly turning the swab for approximately 10 s. 

Cattle were only sampled with vaginal swabs (Sarstedt, Nümbrecht, Germany) as described for small ruminants. The difficult management conditions on the farm made it impossible for more types of samples to be taken (e.g., milk, nasal swab). One positive bovine vaginal swab was included in the MLVA/VNTR typing method to identify the genotype. Calving took place at irregular times during the entire year, e.g., two cows calved at the same day in March 2018. 

It was always the same animals that were sampled with the above-described methods and no new animals were brought onto the farm during the entire study period. At the beginning of the study, the stocking densities in the stables were 23.2 m^2^ per livestock unit (LSU) for goats, 20.3 m^2^/LSU for sheep and 18.5 m^2^/LSU for cattle.

Dust and straw bedding samples were taken from each stable to estimate the *C. burnetii* contamination of the animals’ housings. For that purpose, the same dry sterile swab (Sarstedt, Nümbrecht, Germany) was used as described for the above samples. The dry sterile swab was rolled over the same windowsill of each stable for 1 m to collect stable dust. Additionally, a second dry sterile swab was rolled over the straw bedding for 2 m in the middle of each stable. These environmental specimens were taken every three to four weeks from January 2018 until April 2018 and from November 2018 until April 2019. During the summer and autumn months (May until November 2018) sheep and goats were on separate pastures with no direct contact. The cattle were housed indoors and their barn was mucked out irregularly during the whole study period. At the beginning of May 2018, manure from the sheep and goat shed was removed. Cleansing and disinfection of the animals’ housings were never performed. All swabs and milk samples were stored at −18 °C until laboratory examination. 

The farm is located in the middle of a small village with about 2000 residents. Hence, all ruminants on the farm were vaccinated twice with an inactivated *C. burnetii* phase I vaccine (Coxevac^®^, Ceva Santé Animale, Libourne, France) three weeks apart in January and February 2018 to reduce the risk for the animal owner and local residents. During kidding season 2018, 17 of 22 goats aborted. One goat died at the end of March 2018. The farmer decided to sell most of the goats during summer 2018. Therefore, only seven animals were left by lambing season 2019. Furthermore, two ewes died during summer 2018; therefore, 35 ewes lambed in 2019. After the detection of *C. burnetii* in the goat flock, the farmer did not implement any biosecurity measures (e.g., changing of clothes, disinfection of equipment) when moving between sheds. Though he was informed about the risks for human and animal health.

This study was carried out in accordance with the German animal welfare legislation and the EU Directive 2010/63/EU for animal experiments. Procedures on animals in this study were licensed by the federal state government of Baden-Wuerttemberg under the registration number 35-9185.82/0351.

### 5.2. Molecular Diagnostics

*C. burnetii*-specific DNA fragments in the vaginal, rectal and nasal swabs were detected by amplificating the IS*1111* elements with qPCR. Cycle Threshold (C_t_) values ≤ 45 indicates as positive and C_t_ values > 45 as negative. The details of the used PCR method are published elsewhere [32]. Initially, each animal was detected as positive by using a commercial qPCR (LSI VetMAX^TM^ *C. burnetii* Absolute Quant Kit, Life Technologies GmbH, Darmstadt, Germany) targeting IS*1111* as well. Both methods showed similar results (e.g., due to sensitivity). The limit of detection for both qPCRs is 1 genome equivalent (GE) per PCR evaluated with the Nine Mile genome containing 20 copies of IS*1111* per GE. The dust and straw bedding samples were examined with the same commercially available qPCR (LSI VetMAX^TM^ *C. burnetii* Absolute Quant Kit, Life Technologies GmbH, Darmstadt, Germany). The manufacturer indicates C_t_ values ≤ 45 as positive and C_t_ values > 45 as negative. A plasmid DNA quantification standard (3 × 10^7^ GE copies per mL) is included in the commercial qPCR kit. For the in-house qPCR, we used a plasmid DNA standard (10^8^ GE copies per µL; TIB MOLBIOL Syntheselabor GmbH, Berlin/Heidelberg, Germany). The standard curves were calculated by the MxPro qPCR software version V 4.10 and the Agilent Aria qPCR software version V 1.7 (Agilent Technologies, Santa Clara, CA, USA), respectively. Subsequently, we could use these standard curves to estimate the amount of GE in different samples on the basis of the detected C_t_ value. The amounts of GE were reported as GE per mL due to the initial processing of the swabs in 1 mL phosphate-buffered saline (PBS) according to the qPCR test protocol. 

DNA isolated from vaginal swabs from three sheep, two goats and one cattle were used to conduct the MLVA/VNTR typing method as previously described by Frangoulidis et al. [32]. In brief, isolated DNA was subjected to multiplex PCRs in a total volume of 25 µL containing 1 × Qiagen Multiplex PCR Mix (Qiagen, Hilden, Germany) in a GeneAmp PCR System 9700 (Applied Biosystems, Darmstadt, Germany) thermocycler. The 14 primers are listed elsewhere [32]. Initial denaturation at 95 °C for 15 min was followed by 35 cycles consisting of denaturation at 94 °C for 30 s, primer annealing at 52 °C for 90 s and elongation at 72 °C for 1 min. The final extension step was carried out at 60 °C for 30 min. All amplification products were diluted 1:10 in distilled water, subjected to the Qiagen 50 µL of water. Two microliters of purified amplicons and 3.5 µL of size standard GENESCAN™ 1200LIZ (Applied Biosystems, Darmstadt, Germany) diluted 1:15 in 16.5 µL of HIDI formamide (Applied Biosystems, Darmstadt, Germany) were mixed and denatured for 5 min at 95 °C. The separation of PCR fragments was performed on an ABI 3700 DNA sequencer (Applied Biosystems, Darmstadt, Germany) using the standard GeneScan module. The various tandem repeat loci were analyzed using the nomenclature described by Frangoulidis et al. [32]. 

Isolated DNA from one ovine vaginal swab was used for whole genome sequencing. This sample was also analyzed with the above-mentioned MLVA/VNTR typing method. The library for the sequencing was prepared using the NEBNext^®^ Ultra™ II FS DNA Library Prep Kit for Illumina (New England BioLabs, Frankfurt/Main, Germany) according to the protocol for large fragment sizes > 550 bp but with a minimal fragmentation time of only 30 s. Afterwards, the library was sequenced on an Illumina MiSeq device using the MiSeq Reagent Kit v3 (2 × 300 bp). The sequenced reads were then de novo assembled using SPAdes [47] version 3.13. Finally, the contigs were polished using Pilon version 1.23 [48] in order to determine the number of IS*1111* elements, blastn of the BLAST + package [49] was used to align the conserved IS*1111* insertion sequence against the assembled contigs. All contig ends aligning to the IS*1111* insertion sequence were counted half; contigs fully covered by the insertion sequence were ignored. Consequently, with the known number of IS*1111* copies it was possible to estimate the number of GE in each sample. 

### 5.3. Statistical Analyses

A transformation of the calculated quantities of *C. burnetii* GE/mL was performed by the addition of 1 and base 10 logarithms. Therefore, values of 0 indicate negative samples. Statistical analyses take an alpha level of 0.05 as a basis. Normal distribution was assessed by the Shapiro–Wilk test, Q-Q plots and histograms. The homogeneity of variance was assessed by Levene’s test. Due to the assumption violation of parametric tests in several cases, non-parametric tests are reported. The symmetry of the distribution of differences for paired data was assessed by histograms and symmetry tests. Due to the non-symmetric distribution of differences in some cases, sign tests were performed instead of Wilcoxon signed rank sum tests. Not all comparisons conducted by Wilcoxon rank sum test showed an identical distribution in shape, visualized by histograms. Wilcoxon rank sum tests were conducted for analyses of differences in *C. burnetii* GE/mL concentration for each date in 2018 between species, respectively, for vaginal, rectal, milk and nasal swabs. Data of nasal swabs in March 2018 showed significantly different variances between species and is analyzed descriptively. *p*-values were adjusted by Bonferroni correction, respectively, in each specimen. These analyses were also conducted by Mood’s Median tests showing similar results. Therefore, only results of Wilcoxon rank sum tests are reported.

Differences in frequencies of positive and negative samples between species were analyzed by chi^2^-test with Yates correction if expected frequencies for each condition exceeded five, otherwise fisher’s exact tests were calculated. These analyses were performed separately for each specimen, separately for April 2018 and February 2019 in vaginal, rectal and nasal data, respectively. Milk data were analyzed separately for February 2018 and March 2018. *p*-Values were adjusted with Bonferroni correction for each specimen.

Differences in *C. burnetii* GE/mL concentration within a species for each date in 2018 between rectal and vaginal swabs were analyzed using sign tests with Bonferroni correction for each species, respectively.

Changes in *C. burnetii* GE/mL concentration over dates of measurement in 2018, respectively, for each species separately for vaginal, rectal and nasal data were analyzed by Friedman tests. Sign tests with Bonferroni correction were used for further analyses after significant Friedman test results. Differences in milk specimens from February 2018 to March 2018 were examined by sign tests for each species.

Due to a leak of variance and missing samples, data from February 2019 were excluded from several analyses. Furthermore, data from February 2019 represent a new lambing period. Therefore, these data cannot be included in chronological comparisons. All statistical analyses were performed by the statistical software R, Version 4.0 [50].
